# First-Line Platinum-Based Chemotherapy with Novel Systemic Agents for Unresectable or Metastatic Thymic Epithelial Tumors: A Systematic Review and Meta-Analysis

**DOI:** 10.1016/j.jtocrr.2026.100991

**Published:** 2026-03-21

**Authors:** Gabriela Barbosa e Silva, Lorrany Larisse Costa Rodrigues, Bianca Gonzaga Freitas, Leticia Peixoto Gomes, Mariana Macambira Noronha, Filipe Luís Vasconcelos Visani

**Affiliations:** a Department of Medicine, Oncoclínicas&Co, Rio de Janeiro, Brazil; b Department of Medicine, Central University of Del Paraguay, Paraguay; c Department of Medicine, University Mauricio de Nassau, Sergipe, Brazil; d Department of Medicine, Catholic University of Pernambuco, Pernambuco, Brazil; e Department of Medicine, Federal University of Ceará, Fortaleza, Brazil; f Department of Medicine, Oncoclínicas&Co, Bahia, Brazil

**Keywords:** Thymic epithelial tumors, Thymic carcinoma, Thymomas, Immunotherapy, Antiangiogenic

## Abstract

**Background:**

Thymic epithelial tumors, including thymomas and thymic carcinomas (TC), are rare thoracic malignancies with poor prognosis in advanced stages. Platinum-based chemotherapy (CT) remains the first-line standard with limited efficacy. Novel systemic therapies, such as immune checkpoint inhibitors (ICIs) and antiangiogenic agents, have exhibited promise in refractory disease.

**Methods:**

A systematic search of PubMed, EMBASE, and Cochrane was performed for studies including patients with unresectable, recurrent, or metastatic thymic epithelial tumors treated with platinum-based CT combined with ICIs or antiangiogenic agents. Meta-analyses were conducted using a random-effects model, with heterogeneity assessed through the *I*^*2*^ statistic and Cochran’s Q test.

**Results:**

Nine studies encompassing 208 patients were included, of which 93.7% had TC. The data set comprised five phase II trials and four retrospective cohorts. The pooled 12-month overall survival rate was 96.08% (95% confidence interval [CI]: 84.80–99.08), and 12-month progression-free survival was 56.68% (95% CI: 45.82–70.44). The median duration of response and median progression-free survival were 11.96 (95% CI: 6.32–22.64) months and 16.22 (95% CI: 10.30–25.52) months, respectively, with a pooled objective response rate of 57.69% (95% CI: 43.99–70.3). The objective response rate was 68.50% for antiangiogenic and 51.19% for ICIs combinations. Any-grade treatment-related adverse events occurred in 88.65%, grade 3 or higher in 30.22%, and grade 3 or higher immune-related adverse events in 11.62%, with treatment discontinuation in 13.55%.

**Conclusion:**

Platinum-based CT combined with ICIs or antiangiogenic agents exhibits promising efficacy and manageable toxicity, particularly in TC. Evidence in thymomas remains limited, and severe immune-related toxicity warrants caution. Randomized trials are needed for confirmation.

## Introduction

Thymic epithelial tumors (TETs) are rare and heterogeneous neoplasms of the anterior mediastinum. Thymomas (T) are the most frequent subtype, with an estimated incidence of approximately two cases per million annually in the United States, whereas thymic carcinomas (TC) are less common, occurring at a rate of 0.48 per million.^1^

The WHO classifies TETs according to histologic, morphologic, and molecular features into T subtypes A, AB, B1, B2, and B3 groups, and TC, which typically display squamous differentiation. TC exhibits more aggressive biology with higher metastatic potential, and more than 70% of cases are diagnosed at advanced stages.^2^ Five-year survival rates reflect this disparity, reaching approximately 90% for T, whereas for TCs they decline to 51% to 63% in stage III, 24% to 42% in stage IVA, and 17% to 30% in stage IVB disease.^3^, ^4^, ^5^

For unresectable, recurrent, or metastatic disease, international guidelines recommend platinum-based combination chemotherapy (CT) as the standard first-line systemic treatment. The Eastern Cooperative Oncology Group trial reported an objective response rate (ORR) of 43% in T and 22% in TCs with carboplatin-paclitaxel. However, despite this response rate, most patients relapse within 1 year, and no standard regimen is established for second-line or later settings, in which outcomes remain poor.^5^, ^6^, ^7^

In recent years, novel systemic approaches have been investigated in advanced thymic tumors, including antiangiogenic agents and immune checkpoint inhibitors (ICIs). Multikinase inhibitors such as lenvatinib and regorafenib have exhibited activity after platinum-based chemotherapy.^8^^,^^9^ Similarly, ICIs such as pembrolizumab have exhibited antitumor responses in refractory disease, though immune-related toxicity remains a concern.^10^^,^^11^ These findings support ongoing efforts to integrate these agents into first-line treatment strategies.

In this context, two previous meta-analyses have evaluated systemic therapies for advanced TETs. Arunachalam et al.^12^ summarized outcomes of various agents in pretreated TC, whereas Remon et al.^13^ reported modest efficacy and notable immune-related toxicity with ICIs. Building on these findings, the present meta-analysis investigates the efficacy and safety of platinum-based CT combined with ICIs or antiangiogenic agents in the first-line setting, in which evidence remains scarce because of the rarity of the disease.

## Material and Methods

This systematic review was conducted in accordance with the Preferred Reporting Items for Systematic Reviews and Meta-Analyses guidelines and the Cochrane Handbook for Systematic Reviews of Interventions (Supplementary Table 1).^14^^,^^15^ The review protocol was registered in the International Prospective Register of Systematic Reviews (PROSPERO) (registration number: CRD420251047752).

### Eligibility Criteria and End Points

The inclusion criteria for this meta-analysis were as follows: (1) adult patients (≥18 y) with histologically confirmed TETs, including TC or T, classified according to the WHO histologic classification (fifth Edition)^2^; (2) disease presentation as locally advanced, unresectable, recurrent, or metastatic, corresponding to Masaoka-Koga stage III to IVB or TNM stage IV; (3) not amenable to definitive or curative treatment with surgery, radiotherapy, or CT; (4) platinum-based CT in combination with ICIs or antiangiogenic agents; and (5) previous adjuvant or neoadjuvant therapy allowed, provided it had been completed at least 6 months before study inclusion.

Studies were excluded if they were as follows: (1) case reports or case series with fewer than 10 patients; (2) included previous CT for metastatic TETs; (3) included mixed tumors without separate outcome reporting for TET; or (4) had mixed treatment populations in which outcomes were not reported separately for each treatment modality.

The primary end points were 12-month overall survival (OS) and progression-free survival (PFS) rates, median PFS (mPFS), median duration of response (mDOR), objective response rate (ORR), complete response (CR), partial response (PR), and stable disease. Secondary end points included safety outcomes, specifically the incidence of any-grade treatment-related adverse events (TRAEs), grade 3 or higher (≥3) TRAEs, grade ≥3 immune-related adverse events (irAEs), and treatment discontinuation because of toxicity. Outcome definitions are detailed in Supplementary Table 3

### Search Strategy

From inception to May 26, 2025, a systematic search was conducted across PubMed, EMBASE, and Cochrane. The search strategy included predefined terms related to disease (e.g., T, TC) and interventions (e.g., CT, ICIs, ramucirumab, or bevacizumab), with the full search strategies for all databases detailed in Supplementary Table 2. After duplicate removal, two independent reviewers (L.L.C.R. and G.B.S.) screened titles and abstracts, with potentially relevant reports undergoing full-text evaluation. Discrepancies were resolved by a third reviewer (B.G.F.). Data extraction was independently performed by two authors (B.G.F. and G.B.S.). The following variables were collected: study reference (author and y), trial design, country, population, median age (y), CT regimen, sex distribution, follow-up duration (mo), Masaoka stage, presence of extrathoracic metastases, and previous therapies.

### Quality Assessment

The risk of bias (RoB) for randomized controlled trials (RCTs) was evaluated using version 2 of the Cochrane RoB tool (RoB 2).^16^ For nonrandomized studies, RoB was assessed using the RoB in Nonrandomized Studies of Interventions (ROBINS-I) tool.^17^ Two reviewers (F.L.V.V. and L.P.G.) independently conducted the assessments, with any discrepancies resolved by consensus and arbitration from a third reviewer (B.G.F.). The certainty of the evidence was evaluated by two independent authors (G.B.eS. and L.P.G.) using the Grading of Recommendations Assessment, Development, and Evaluation (GRADE) approach.

Given the limited number of included studies and the considerable clinical and methodological heterogeneity, formal assessment of publication bias (e.g., funnel plot inspection or Egger’s regression test) was not methodologically appropriate. To mitigate potential publication and selective-reporting biases, we performed a comprehensive search strategy that included trial registries, full-text publications, and conference proceedings.

### Statistical Analysis

Statistical analyses were performed using R software (version 2022.12.0+353; R Core Team, Vienna, Austria) using the meta package.^18^ Statistical significance was defined as a two-sided *p* value less than 0.05. Heterogeneity was assessed using the *I*^*2*^ statistic and Cochran’s Q test, and interpreted as low (<25%), moderate (25%–75%), or high (>75%).

Sensitivity analyses were conducted using meta-analyses of proportions, including subgroup analyses according to drug class (ICIs versus antiangiogenic agents) and study design (retrospective versus prospective studies). A leave-one-out approach was also performed, in which the meta-analysis was repeated sequentially, excluding one study at a time, to assess the robustness of the pooled estimates.

Meta-analyses were conducted using a random-effects model with the generic inverse variance method, and pooled proportions were reported with 95% confidence intervals (CIs). For binary outcomes, a proportional meta-analysis was performed using a logit transformation when individual study proportions were less than 0.2 or greater than 0.8. In cases with zero events or proportions equal to 1.0, the Freeman-Tukey double-arcsine transformation was applied.

For time-to-event outcomes, a single-arm meta-analysis was used to pool median survival times, as eligible studies did not report hazard ratios or fixed-time survival rates because of their single-arm design. The SE for mPFS was calculated as:

$$SE=\frac{\ln\;\left(Upper95\%CI\right)-\ln\left(Lower95\%CI\right)}{2\times 1,96}$$

Log-transformed median survival times were then pooled using a random-effects model and back-transformed by exponentiation. Studies reporting mPFS without corresponding 95% CIs were excluded from these analyses.

### Ethics Statement

This study represents a secondary analysis of previously published data and did not involve direct patient contact or access to identifiable individual-level information. Therefore, institutional review board approval and informed consent were not required.

## Results

### Study Selection and Baseline Characteristics

**Figure 1:**
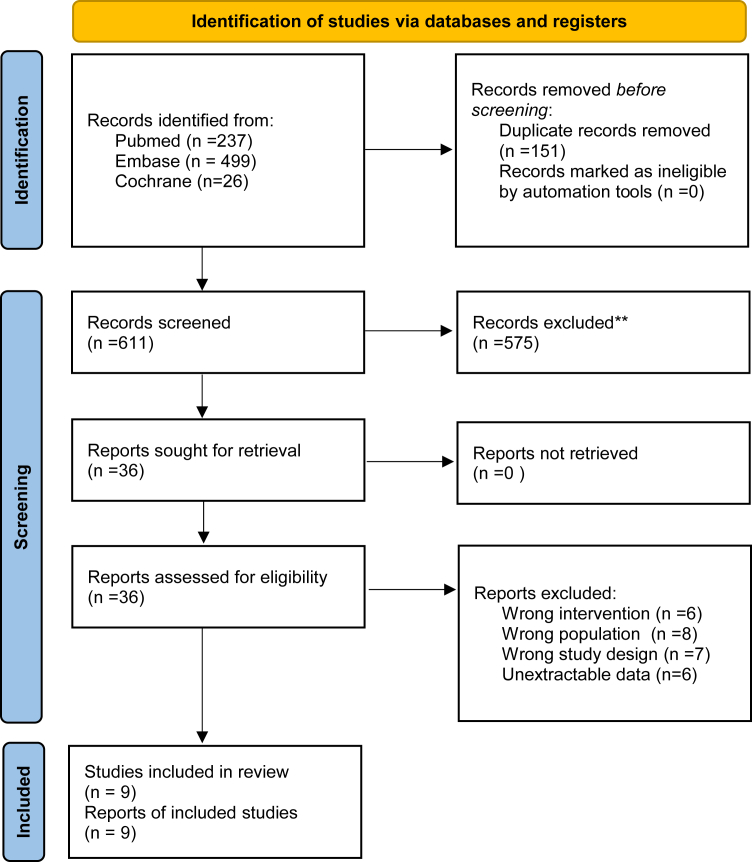
PRISMA flow diagram of study screening and selection. PRISMA, Preferred Reporting Items for Systematic Reviews and Meta-Analyses.

As illustrated in Figure 1, the initial search identified 762 records. After removing duplicates and ineligible studies, 36 full-text articles were assessed for eligibility, and nine met the inclusion criteria,^19^, ^20^, ^21^, ^22^, ^23^, ^24^, ^25^, ^26^, ^27^ comprising five phase II trials and four observational cohorts, totaling 208 patients included in the quantitative synthesis.

The included studies evaluated two antiangiogenic regimens with ramucirumab, one with endostar,^20^, ^21^, ^22^ and six ICIs combinations (atezolizumab, toripalimab, pembrolizumab, tislelizumab, an unspecified programmed cell death protein 1 inhibitor, and a pooled ICIs cohort).^19^^,^^23^, ^24^, ^25^, ^26^, ^27^ Six studies enrolled patients with recurrent, treatment-naive metastatic disease.^19^, ^20^, ^21^, ^22^, ^23^^,^^27^ Previous surgery or radiotherapy was reported in three studies,^19^^,^^22^^,^^23^ and two included patients who had received neoadjuvant or adjuvant CT.^21^^,^^23^

Baseline characteristics are summarized in Table 1. Most patients were male (86.7%), with a median age of 60.4 years. TC accounted for 93.7% of cases—77.2% with squamous histologic subtype—whereas T represented 6.2%. The median follow-up ranged from 15.3 to 55 (median 24.8) months.

**Table 1:** Characteristics of the Studies Included in the Meta- Analysis

Study	Population	Novel Systemic Drug	Male	Age^a^	Study Design	CT	Follow-Up^b^	Total	Subtype		Masaoka Stage		Thymic Squamous Cell		Extrathoracic Metastases	Previous Therapy		Country
									TC	Thymoma	III	IVA/IVB	Yes	No		Yes	No	
Zhang et al.^19^ 2024	Unresectable	Pembrolizumab (N = 8) Sintilimab (N = 8) Tislelizumab (N = 15) Camrelizumab (N = 5) Atezolizumab (N = 2)	24 (61.5%)	NA	Coorte retrospective	TP or CAP or other platinum CT^c^	34.9	39	39 (100%)	0	9 (23.1%)	30 (76.9%)	33 (84.6%)	6 (15.4%)	13 (33.3%)	29^d^ (74.4%)	10 (25.6%)	People’s Republic of China
Proto et al.^20^ 2024	Advanced, recurrent or metastatic	Ramucirumab	25 (71.4%)	60.8^e^	Phase II	TP^b^	31.6	35	35 (100%)	0	0	35 (100%)	30 (85.7%)	5 (14.3%)	31 (88.6%)	NA	NA	Italy
Tsao et al.^21^ 2024	Advanced, recurrent or metastatic	Ramucirumab	7 (88%)	68.2	Randomized phase II	Ramucirumab + TP	16.7	8	6 (75%)	0	NA	NA	6 (75%)	2 (25%)	3 (38%)	2^f^	6	USA
Wang et al.^22^ 2018	Metastatic	Endostar	24^e^ (53.3%)	50^e^	Coorte retrospective	Gemcitabine + cisplatin	55	24	14 (31.1%)	10 (22.2%)	10^e^ (22.2%)	35^e^ (77.8%)	21^e^ (46.6%)	8^e^ (17.7%)	37^e^ (82.2%)	20^d^^,^^e^ (44.4%)	0	People’s Republic of China
Shukuya et al.^23^ 2025	Metastatic or recurrent	Atezolizumab	29 (60%)	67.5	Phase II	TP	15.3	48	48 (100%)	0	1 (2%)	35 (72.9%)	34 (71%)	14 (29.1%)	NA	25^d^ (52%) 3^f^ (6%)	32 (66.6%)	Japan
Zhu et al.^24^ 2025	Advanced	Toripalimab	16 (66.7%)	55	Phase II	TP	15.3	24	24 (100%)	0	N	NA	NA	NA	NA	0	24	People's Republic of China
Liu et al.^25^ 2023	Advanced	PD-1 inhibitor^g^	30^e^ (62.5%)	54^e^	Coorte retrospective	Platinum-based CT^c^	NA	19	19 (100%)	0	NA	NA	38^e^ (79.2%)	10 (20.8%)	NA	NA	NA	People's Republic of China
Andreas et al.^26^ 2023	Metastatic	Pembrolizumab	9^e^ (52.9%)	67^e^	Coorte retrospective	Platinum-based CT^c^	24.8	6	6 (100%)	0	NA	NA	11^e^ (65%)	6^e^ (35%)	NA	NA	NA	Australia
Zhang et al.^27^ 2024	Unresectable or recurrent	Tislelizumab	4 (50%)	61^e^	Phase II	Thymoma: CAP TC: TP	NA	8^h^	5 (62.5%)	3 (37.5%)	NA	NA	NA	NA	NA	NA	NA	People's Republic of China

CAP, cyclophosphamide, doxorubicin, and cisplatin; CT: chemotherapy; NA: not available; RT: radiotherapy; TC, Thymic carcinoma; TP: paclitaxel and cisplatin.

*a* Median.

*b* Months.

*c* Medication drug not reported.

*d* Previous RT and/or surgery.

*e* Only overall sample data available.

*f* Previous neo- or adjuvant CT.

g The specific PD-1 inhibitor was not specified in the original study.

*h* Five patients were evaluated for efficacy.

Platinum-based doublets represented the predominant CT backbone across the included studies, most frequently combining carboplatin with paclitaxel. Zhang et al.^19^ investigated multiple platinum-containing regimens, including CAP (cyclophosphamide, doxorubicin, and cisplatin (n = 8) and other unspecified combinations (n = 4). Wang et al.^22^ evaluated gemcitabine plus cisplatin, whereas Zhang et al.^27^ administered CAP for T and carboplatin-paclitaxel for TC. Two conference abstracts reported platinum-based CT without further regimen specification.^25^^,^^26^

When reported, most patients are presented with advanced or metastatic disease. Stage IVB accounted for 61.5% of cases in Zhang et al.,^19^ 85.7% in Proto et al.,^20^ and 52% in Shukuya et al.^23^ Programmed death-ligand 1 expression was infrequently assessed, with tumor proportion score greater than or equal to 1% were observed in 38.5% and 44% of patients in Zhang et al.,^19^ and Shukuya et al.,^23^ respectively.

### Pooled Efficacy Analysis

**Figure 2:**
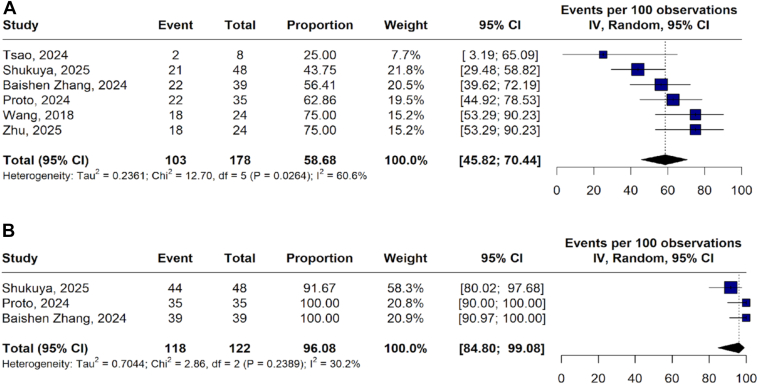
Twelve-month progression-free survival (*A*) and 12-month overall survival (*B*).CI, confidence interval; IV, inverse variance.

The pooled 12-month PFS rate was 56.68% (95% CI: 45.82–70.44; *I*^*2*^ = 60.6 (Fig. 2*A*), and the 12-month OS rate was 96.08% (95% CI: 84.80–99.08; *I*^*2*^ = 30.2) (Fig. 2*B*).

The median PFS and mDOR were 16.22 (95% CI: 10.30–25.52; *I*^*2*^ = 91.6%) months and 11.96 (95% CI: 6.32–22.64; *I*^*2*^ = 23.8%) months, respectively (Supplementary Figs. 1A and 2).

**Figure 3:**
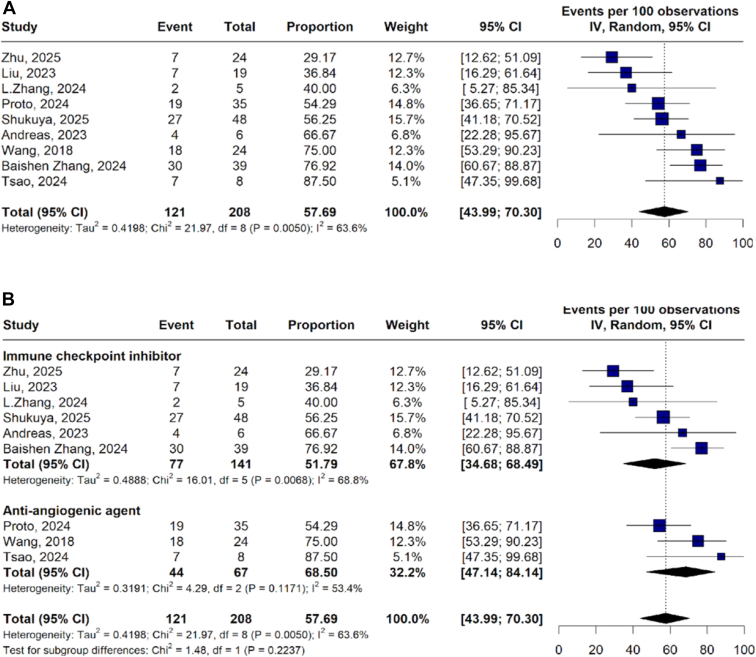
Objective response rate (*A*) and subgroup analysis by therapeutic regimen (*B*). CI, confidence interval; IV, inverse variance.

The pooled ORR was 57.69% (95% CI: 43.99–70.30; *I*^*2*^ = 63.6%) (Fig. 3*A*). Among response categories, CR occurred in 0.25% of patients (95% CI: 0.00–3.70; *I*^*2*^ = 51.7%), PR in 56.23% (95% CI: 41.38–70.05; *I*^*2*^ = 63.6%), and stable disease in 35.22% (95% CI: 26.68%–44.82%; *I*^*2*^ = 33.7%) (Supplementary Figs. 3–5).

### Subgroup and Sensitivity Analyses

In subgroup analysis by drug class, ICIs-based combinations achieved a longer mPFS of 19.45 (95% CI: 8.94–42.32; *I*^*2*^ = 91%) months compared with 13.64 (95% CI: 6.82–27.28; *I*^*2*^ = 94.4%) months for antiangiogenic regimens (Supplementary Fig. 1B). Stratified by study design, mPFS was 24.64 (95% CI: 13.66–44.43; *I*^*2*^ = 81.1%) months in retrospective cohort studies and 12.74 (95% CI: 7.69–21.11; *I*^*2*^ = 86%) months (Supplementary Fig. 1C) in prospective trials.

The pooled ORR was 68.50% (95% CI: 47.14–84.14; *I*^*2*^ = 53.4%) for antiangiogenic regimens and 51.79% (95% CI: 34.68–68.5; *I*^*2*^ = 68.49%) for ICIs-based therapies (Fig. 3*B*). The rates of PR were higher for antiangiogenic agents 68.50% (95% CI: 47.14–84.14; *I*^*2*^ = 53.4%) than ICIs 47.15% (95% CI: 27.86–67.33; *I*^*2*^ = 69.8%) (Supplementary Fig. 4B). The rates of stable disease were comparable between ICIs 37.71% (95% CI: 25.25–52.02; *I*^*2*^ = 44.8%) versus antiangiogenic 30.72% (95% CI: 18.20–46.92; *I*^*2*^ = 28.3%) (Supplementary Fig. 5B).

The 12-month PFS rate was comparable among therapeutic classes, reaching 57.33% (95% CI: 39.51–73.42; *I*^*2*^ = 66.9%) for ICIs-based combinations and 58.77% (95% CI: 33.06–80.44; *I*^*2*^ = 63.2%) (Supplementary Fig. 6A) for antiangiogenic regimens.

Leave-one-out sensitivity analyses were performed both in the overall mPFS analysis and in the CT plus ICI subgroup. In the overall analysis, sequential exclusion of individual studies did not materially change the pooled estimates (Supplementary Fig. 7). In the ICI subgroup, exclusion of Zhang et al.^19^ reduced the pooled estimate to 14.72 months, whereas removal of Shukuya et al.^23^ increased mPFS to 27.75 (95% CI: 18.48–41.67; *I*^*2*^ = 38.9%) months (Supplementary Fig. 8).

### Safety Outcomes

**Figure 4:**
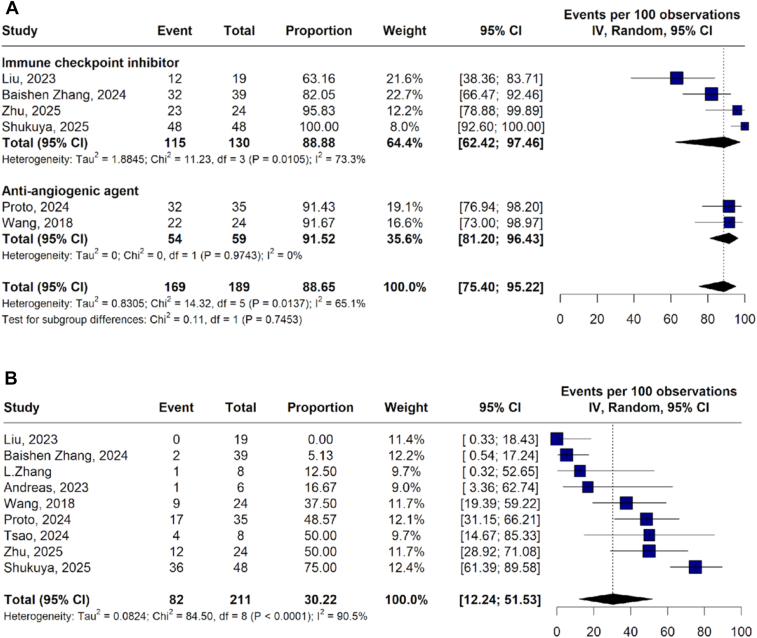
Any-grade TRAEs subgroup analysis by therapeutic regimen (*A*) and grade 3 or higher TRAEs (*B*). CI, confidence interval; IIV, inverse variance; TRAEs, treatment-related adverse events.

The pooled incidence of any-grade TRAEs was 88.65% (95% CI: 75.40–95.22; *I*^*2*^ = 65.1%), with comparable rates among antiangiogenic combinations 91.52% (95% CI: 81.20–96.43; *I*^*2*^ = 0%) and ICIs 88.88% (95% CI: 62.42–97.46; *I*^*2*^ = 73.3%) (Fig. 4A).

The most frequent any-grade toxicities included peripheral neuropathy 53.34%, anemia 41.07%, neutropenia 36.21%, thrombocytopenia 24.13%, fatigue 23.53%, and alanine aminotransferase elevation 17.3% (Supplementary Fig. 9).

Grade ≥3 TRAEs occurred in 30.22% of patients (95% CI: 12.21–51.53; *I*^*2*^ = 90.5%) (Fig. 4*B*). Incidence was higher with antiangiogenic combinations 44.63% (95% CI: 32.38–57.19; *I*^*2*^ = 0%) compared with ICIs-based regimens 23.03% (95% CI: 2.45–52.99; *I*^*2*^ = 93.8%) (Supplementary Fig. 10).

Treatment discontinuation because of toxicity was reported in 13.55% (95% CI: 8.44–21.05; *I*^*2*^ = 12.6%), with higher rates among antiangiogenic combinations 18.77% (95% CI: 9.66–33.30; *I*^*2*^ = 0%) than among ICIs-based regimens 9.46% (95% CI: 4.11–20.28; *I*^*2*^ = 24%) (Supplementary Fig. 11A and B).

Any-grade of irAEs occurred in 40.74% of patients (95% CI: 9.06–82.60; *I*^*2*^ = 77.7%), most frequently hypothyroidism (11.3%) and pneumonitis (8.2%). Grade ≥3 irAEs were observed in 11.25% (95% CI: 0.42–29.69; *I*^*2*^ = 82.8%) (Supplementary Fig. 12A and B).

### Quality Assessment and Certainty of Evidence

In the RoB assessment, seven of eight nonrandomized studies were judged to have a serious RoB according to ROBINS-I, whereas the single RCT was rated as having some concerns using the RoB two tool (Supplementary Fig. 13A and B). GRADE assessments of the certainty of evidence for the primary outcomes are presented in Supplementary Table 4.

## Discussion

This systematic review and meta-analysis present the most extensive evaluation to date of platinum-based CT combined with novel agents as first-line therapy for TETs. Across nine studies including 208 patients, the pooled ORR was 57.7%, driven predominantly by PRs, whereas CRs were uncommon, occurring in only 0.25% of cases. The mDOR was 11.96 months, and the mPFS reached 16.22 months, both longer than historical benchmarks with CT alone, in which PFS seldom exceeds 5 to 8 months.^4^ Our findings suggest that regimens combining antiangiogenic agents with platinum-based CT were associated with numerically higher ORR, whereas chemoimmunotherapy combinations appeared to achieve longer mPFS.

Recent prospective studies have provided early clinical evidence supporting this approach. The RELEVENT (phase II trial of ramucirumab, carboplatin, and paclitaxel in previously untreated TC/ B3 T with carcinoma component) trial enrolled 41 patients with previously untreated advanced TC and assessed ramucirumab plus carboplatin and paclitaxel, reporting an independent central review–confirmed ORR of 57.6%, a mPFS of 18.1 months, and a median OS of 43.8 months.^20^ These results led to the inclusion of this regimen as a preferred option for TC in the 2025 National Comprehensive Cancer Network guidelines.^5^ The MARBLE (multicenter phase II trial of atezolizumab plus carboplatin and paclitaxel in advanced or recurrent TC) trial (n = 48; previously untreated TC, 71% squamous histology) evaluated atezolizumab combined with platinum-based CT and reported an ORR of 56%, a mPFS of 9.6 months, and a disease control rate of 98%, with grade ≥3 TRAEs in 17% of patients.^23^ Although the randomized phase II S1701 (trial of carboplatin–paclitaxel with or without ramucirumab in locally advanced, recurrent, or metastatic TC) (n = 21; locally advanced or metastatic TC) was closed early and underpowered, it suggested a doubling of ORR in the ramucirumab plus CT arm compared with CT alone, along with a favorable PFS trend.^21^

In the post-platinum setting, the REMORA (multicenter phase II trial of lenvatinib in advanced or metastatic TC) trial evaluated lenvatinib in 42 patients with previously treated TC and reported an independent review committee–assessed ORR of 38%, a mPFS of 9.3 months, and a median OS of 28.3 months; notably, higher relative dose intensity was associated with longer survival and higher response rates.^8^ Sunitinib and regorafenib have also exhibited activity in pretreated disease, further supporting the rationale for earlier integration of antiangiogenic therapy.^9^^,^^28^

The biologic rationale for these combinations is supported by previous research. CT has been found to induce immunogenic cell death and enhance antigen presentation, whereas antiangiogenic therapy may normalize tumor vasculature, alleviate hypoxia, and promote immune cell infiltration.^29^^,^^30^ These mechanisms, combined with the dependence of many TETs on the VEGF signaling pathway, may help explain the favorable signals observed with chemoimmunotherapy or chemoantiangiogenic regimens.^10^^,^^20^^,^^21^^,^^23^

Safety findings were consistent with known class effects. Severe TRAEs occurred in approximately 45% of patients receiving antiangiogenic combinations and approximately 42% of those treated with ICIs. Hematologic toxicities, neuropathy, and fatigue predominated with antiangiogenics, whereas hypothyroidism (11.32%) and pneumonitis (8.20%) were the most frequent irAEs. However, our analysis is predominantly weighted toward TC (93.7%), and the risks of severe irAEs in T, including myasthenia gravis, myocarditis, and myositis, may not be fully represented.^3^

These results highlight both the promise and the uncertainty of combination strategies in TETs. The signals of improved outcomes are encouraging, particularly for antiangiogenic regimens, which consistently produced the highest response rates in our analysis. Still, the moderate degree of heterogeneity means that these findings must be regarded as hypothesis-generating. Early-phase studies such as PECATI (multicenter, open-label, single-arm phase II study of pembrolizumab plus lenvatinib in pretreated B3 T and TC) and STYLE (phase II study of sunitinib in patiets with B3 T and TC in second or later lines), and ongoing trials testing triplet strategies that combine CT, antiangiogenic agents, and ICIs (including tislelizumab-based regimens), represent promising next steps.^31^^,^^32^ However, predictive biomarkers such as programmed death-ligand 1 expression and tumor mutational burden have exhibited inconsistent associations with response. Given the rarity of TETs and the challenges in conducting adequately powered RCTs, multinational collaborations, pragmatic phase II designs, and harmonized reporting will be essential to determine which combinations can be established as new first-line standards.^33^

This meta-analysis evaluates novel systemic therapies as first-line treatment in locally advanced or metastatic TETs. However, several limitations merit consideration. Most included studies were single-arm phase II trials or retrospective cohorts with limited sample sizes, reflecting the rarity of TETs and inherently increasing the risk of selection bias, residual confounding, and publication bias. Considerable clinical and methodological heterogeneity was observed across studies, including differences in patient populations, previous lines of therapy, follow-up duration, and variability in treatment regimens, which may have influenced the pooled estimates. Despite predefined subgroup and sensitivity analyses according to study design and treatment class, substantial heterogeneity persisted, suggesting that between-study variability was multifactorial rather than attributable to a single source.

In the ICI-based subgroup, leave-one-out analyses indicated sensitivity of the pooled mPFS estimate to individual studies, highlighting the instability of summary effects in the context of small, heterogeneous data sets. These findings reinforce the challenges of synthesizing predominantly nonrandomized evidence in rare malignancies. In line with these methodological constraints, the certainty of evidence was rated as very low according to the GRADE framework, and results should, therefore, be interpreted with caution.

In conclusion, this study suggests that the combination of platinum-based CT with ICIs or antiangiogenic agents as first-line therapy for advanced TETs is associated with clinically meaningful antitumor activity, specifically for TC. Evidence for T remains insufficient to support routine use. These results provide a benchmark for future investigations and underscore the need for RCTs to determine the optimal first-line treatment strategy in this rare malignancy.

## CRediT Authorship Contribution Statement

**Gabriela Barbosa e Silva**: Conceptualization, Methodology, Software, Validation, Formal analysis, Data curation, Writing - original draft, Writing - review & editing, Supervision.

**Lorrany Larisse Costa Rodrigues**: Conceptualization, Methodology, Software, Validation, Formal analysis, Data curation, Writing - original draft.

**Bianca Gonzaga Freitas**: Methodology, Validation, Formal analysis, Data curation, Writing - review & editing.

**Leticia Peixoto Gomes**: Methodology, Data curation, Writing - review & editing.

**Mariana Noronha**: Validation, Writing - review & editing.

**Filipe Visani**: Formal analysis, Validation, Writing - review & editing, Supervision.

## Disclosure

The authors declare no conflict of interest.

## Footnotes

Note: To access the supplementary material accompanying this article, visit the online version of the *JTO Clinical and Research Reports* at www.jtocrr.org and at https://doi.org/10.1016/j.jtocrr.2026.100991.
