# Supplementary material for: First-Line Platinum-Based Chemotherapy with Novel Systemic Agents for Unresectable or Metastatic Thymic Epithelial Tumors: A Systematic Review and Meta-Analysis
Source: JTO Clin Res Rep. 2026 Mar 21;7(6):100991. doi: 10.1016/j.jtocrr.2026.100991 (PMC13181280; doi:10.1016/j.jtocrr.2026.100991)

**Supplementary Material**

**First-Line Platinum-Based Chemotherapy with Novel Systemic Agents for Unresectable or Metastatic Thymic Epithelial Tumors: A Systematic Review and Meta-Analysis**

Gabriela Barbosa e Silva^1^, Lorrany Larisse Costa Rodrigues^2^, Bianca Gonzaga Freitas^3^, Leticia Peixoto Gomes^4^, Mariana Macambira Noronha^5^, Filipe Luís Vasconcelos Visani^6^

Summary

**Table 1:** Preferred reporting items for systematic reviews and meta-analyses (PRISMA) checklist for the manuscript (A), and for the abstract (B).

**Table 2.** Full search strategy for each database. (A) Embase; (B) Cochrane; (C) PubMed.

**Table 3.** Outcome definitions.

**Table 4.** Summary of findings and GRADE certainty of evidence assessment for primary outcomes.

**Figure 1.** Median progression-free survival (PFS) (A). Subgroup analysis by therapeutic regimen (B).

**Figure 2.** Median duration of response (mDOR).

**Figure 3.** Complete response (CR).

**Figure 4.** Partial response (PR) (A). Subgroup analysis by therapeutic regimen (B).

**Figure 5.** Stable disease (A). Subgroup analysis by therapeutic regimen (B).

**Figure 6.** 12-month progression-free survival (PFS). Subgroup analysis by drug class (A) and by study design (B).

**Figure 7.** Leave-one-out sensitivity analysis for pooled median progression-free survival (mPFS).

**Figure 8.** Leave-one-out sensitivity analysis of pooled median progression-free survival (mPFS) restricted to ICI-based regimens.

**Figure 9.** Most frequent any-grade treatment-related adverse events (TRAEs).

**Figure 10.** Treatment-related adverse events grade ≥ 3 subgroup analysis by therapeutic regimen.

**Figure 11.** Treatment discontinuation due to toxicity (A). Subgroup analysis by therapeutic regimen (B).

**Figure 12.** Immune-related any-grade treatment-related adverse events (irAEs) (A). Grade ≥ 3 (B).

**Figure 13.** Risk of bias assessed by RoB-2 (A) and ROBINS-I (B).

**Table 1:** Preferred reporting items for systematic reviews and meta-analysis (PRISMA) checklist for the manuscript (A), and for the abstract (B).

**Table 1A.** Manuscript PRISMA checklist.

| **Section and Topic** | **Item #** | **Checklist item** | **Location where item is reported** |
| --- | --- | --- | --- |
| **TITLE** | | |  |
| **Title** | **1** | Identify the report as a systematic review. | **Page 1** |
| **ABSTRACT** | | |  |
| **Abstract** | **2** | See the PRISMA 2020 for Abstracts checklist. | **Table S1B** |
| **INTRODUCTION** | | |  |
| **Rationale** | **3** | Describe the rationale for the review in the context of existing knowledge. | **Page 4** |
| **Objectives** | **4** | Provide an explicit statement of the objective(s) or question(s) the review addresses. | **Page 4** |
| **METHODS** | | |  |
| **Eligibility criteria** | **5** | Specify the inclusion and exclusion criteria for the review and how studies were grouped for the syntheses. | **Page 5, 6** |
| **Information sources** | **6** | Specify all databases, registers, websites, organisations, reference lists and other sources searched or consulted to identify studies. Specify the date when each source was last searched or consulted. | **Pages 5** |
| **Search strategy** | **7** | Present the full search strategies for all databases, registers and websites, including any filters and limits used. | **Page 5 and Table S2** |
| **Selection process** | **8** | Specify the methods used to decide whether a study met the inclusion criteria of the review, including how many reviewers screened each record and each report retrieved, whether they worked independently, and if applicable, details of automation tools used in the process. | **Page 5** |
| **Data collection process** | **9** | Specify the methods used to collect data from reports, including how many reviewers collected data from each report, whether they worked independently, any processes for obtaining or confirming data from study investigators, and if applicable, details of automation tools used in the process. | **Pages 5, 6** |
| **Data items** | **10a** | List and define all outcomes for which data were sought. Specify whether all results that were compatible with each outcome domain in each study were sought (e.g. for all measures, time points, analyses), and if not, the methods used to decide which results to collect. | **Page 6** |
|  | **10b** | List and define all other variables for which data were sought (e.g. participant and intervention characteristics, funding sources). Describe any assumptions made about any missing or unclear information. | **NA** |
| **Study risk of bias assessment** | **11** | Specify the methods used to assess risk of bias in the included studies, including details of the tool(s) used, how many reviewers assessed each study and whether they worked independently, and if applicable, details of automation tools used in the process. | **Pages 6, 7** |
| **Effect measures** | **12** | Specify for each outcome the effect measure(s) (e.g. risk ratio, mean difference) used in the synthesis or presentation of results. | **Pages 7, 8** |
| **Synthesis methods** | **13a** | Describe the processes used to decide which studies were eligible for each synthesis (e.g. tabulating the study intervention characteristics and comparing against the planned groups for each synthesis (item #5)). | **NA** |
|  | **13b** | Describe any methods required to prepare the data for presentation or synthesis, such as handling of missing summary statistics, or data conversions. | **NA** |
|  | **13c** | Describe any methods used to tabulate or visually display results of individual studies and syntheses. | **NA** |
|  | **13d** | Describe any methods used to synthesize results and provide a rationale for the choice(s). If meta-analysis was performed, describe the model(s), method(s) to identify the presence and extent of statistical heterogeneity, and software package(s) used. | **Pages 7, 8** |
|  | **13e** | Describe any methods used to explore possible causes of heterogeneity among study results (e.g. subgroup analysis, meta-regression). | **Page 8, 11** |
|  | **13f** | Describe any sensitivity analyses conducted to assess robustness of the synthesized results. | **NA** |
| **Reporting bias assessment** | **14** | Describe any methods used to assess risk of bias due to missing results in a synthesis (arising from reporting biases). | **NA** |
| **Certainty assessment** | **15** | Describe any methods used to assess certainty (or confidence) in the body of evidence for an outcome. | **NA** |
| **RESULTS** | | |  |
| **Study selection** | **16a** | Describe the results of the search and selection process, from the number of records identified in the search to the number of studies included in the review, ideally using a flow diagram. | **Page 8** |
|  | **16b** | Cite studies that might appear to meet the inclusion criteria, but which were excluded, and explain why they were excluded. | **Table 2 Supplemental** |
| **Study characteristics** | **17** | Cite each included study and present its characteristics. | **Page 8, Table 1, Figure 2** |
| **Risk of bias in studies** | **18** | Present assessments of risk of bias for each included study. | **Pages 10, 11, Figure S1** |
| **Results of individual studies** | **19** | For all outcomes, present, for each study: (a) summary statistics for each group (where appropriate) and (b) an effect estimate and its precision (e.g. confidence/credible interval), ideally using structured tables or plots. | **Pages 9,10, Figures 3,4 Figure S1** |
| **Results of syntheses** | **20a** | For each synthesis, briefly summarise the characteristics and risk of bias among contributing studies. | **Figure S2** |
|  | **20b** | Present results of all statistical syntheses conducted. If meta-analysis was done, present for each the summary estimate and its precision (e.g. confidence/credible interval) and measures of statistical heterogeneity. If comparing groups, describe the direction of the effect. | **Pages 9, 10** |
|  | **20c** | Present results of all investigations of possible causes of heterogeneity among study results. | **Pages 10, 11** |
|  | **20d** | Present results of all sensitivity analyses conducted to assess the robustness of the synthesized results. | **NA** |
| **Reporting biases** | **21** | Present assessments of risk of bias due to missing results (arising from reporting biases) for each synthesis assessed. | **NA** |
| **Certainty of evidence** | **22** | Present assessments of certainty (or confidence) in the body of evidence for each outcome assessed. | **NA** |
| **DISCUSSION** | | |  |
| **Discussion** | **23a** | Provide a general interpretation of the results in the context of other evidence. | **Pages 11, 12** |
|  | **23b** | Discuss any limitations of the evidence included in the review. | **Page 13** |
|  | **23c** | Discuss any limitations of the review processes used. |  |
|  | **23d** | Discuss implications of the results for practice, policy, and future research. | **Page 13** |
| **OTHER INFORMATION** | | |  |
| **Registration and protocol** | **24a** | Provide registration information for the review, including register name and registration number, or state that the review was not registered. | **Page 4** |
|  | **24b** | Indicate where the review protocol can be accessed, or state that a protocol was not prepared. | **Page 4** |
|  | **24c** | Describe and explain any amendments to information provided at registration or in the protocol. | **NA** |
| **Support** | **25** | Describe sources of financial or non-financial support for the review, and the role of the funders or sponsors in the review. | **NA** |
| **Competing interests** | **26** | Declare any competing interests of review authors. | **Page 1** |
| **Availability of data, code and other materials** | **27** | Report which of the following are publicly available and where they can be found: template data collection forms; data extracted from included studies; data used for all analyses; analytic code; any other materials used in the review. | **NA** |

NA: Not available

**1B.** Abstract PRISMA checklist.

| **Section and Topic** | **Item #** | **Checklist item** | **Reported (Yes/No)** |
| --- | --- | --- | --- |
| **TITLE** | | |  |
| **Title** | **1** | Identify the report as a systematic review. | **Yes** |
| INTRODUCTION | | |  |
| **Objectives** | **2** | Provide an explicit statement of the main objective(s) or question(s) the review addresses. | **Yes** |
| METHODS | | |  |
| **Eligibility criteria** | **3** | Specify the inclusion and exclusion criteria for the review. | **Yes** |
| **Information sources** | **4** | Specify the information sources (e.g. databases, registers) used to identify studies and the date when each was last searched. | **Yes** |
| **Risk of bias** | **5** | Specify the methods used to assess risk of bias in the included studies. | **No** |
| **Synthesis of results** | **6** | Specify the methods used to present and synthesise results. | **Yes** |
| RESULTS | | |  |
| **Included studies** | **7** | Give the total number of included studies and participants and summarise relevant characteristics of studies. | **Yes** |
| **Synthesis of results** | **8** | Present results for main outcomes, preferably indicating the number of included studies and participants for each. If meta-analysis was done, report the summary estimate and confidence/credible interval. If comparing groups, indicate the direction of the effect (i.e. which group is favoured). | **Yes** |
| CONCLUSION | | |  |
| **Limitations of evidence** | **9** | Provide a brief summary of the limitations of the evidence included in the review (e.g. study risk of bias, inconsistency and imprecision). | **No** |
| **Interpretation** | **10** | Provide a general interpretation of the results and important implications. | **Yes** |
| OTHER | | |  |
| **Funding** | **11** | Specify the primary source of funding for the review. | **No** |
| **Registration** | **12** | Provide the register name and registration number. | **No** |

**Table 2.** Full search strategy for each database. (A) Embase; (B) Cochrane; (C) PubMed.

1. Embase

| 1 | thymic epithelial tumor | ('thymic epithelial tumor' OR 'tet' OR 'thymic carcinoma' OR 'thymoma' OR 'thymic tumor' OR 'thymic neoplasm' OR 'thymic malignancy' OR 'thymic neoplasm'/exp) |
| --- | --- | --- |
| 2 | disease stage | ('advanced' OR 'unresectable' OR 'metastatic' OR 'recurrent') |
| 3 | platinum-based chemotherapy | ('carboplatin' OR 'cisplatin' OR 'oxaliplatin' OR 'platinum derivative'/exp OR 'paclitaxel' OR 'docetaxel' OR 'doxorubicin' OR 'anthracyclines' OR 'chemotherapy') |
| 4 | novel systemic therapies | ('targeted therapy' OR 'targeted treatment' OR 'targeted agent' OR 'tyrosine kinase inhibitor' OR 'tki' OR 'crizotinib' OR 'axitinib' OR 'lenvatinib' OR 'ramucirumab' OR 'bevacizumab' OR 'cetuximab' OR 'molecularly targeted therapy'/exp OR 'checkpoint inhibitor' OR 'checkpoint blockade' OR 'immune checkpoint therapy' OR 'immunotherapy' OR 'nivolumab' OR 'pembrolizumab' OR 'cemiplimab' OR 'toripalimab' OR 'sintilimab' OR 'camrelizumab' OR 'atezolizumab' OR 'durvalumab' OR 'avelumab' OR 'ipilimumab' OR 'tremelimumab' OR 'immune checkpoint inhibitor'/exp) |
|  |  | #1, #2, #3 AND #4 |

1. Cochrane

| 1 | thymic epithelial tumor | (“Thymic epithelial tumor” OR TET OR “Thymic carcinoma” OR “Thymoma” OR “Thymic tumor” OR “Thymic neoplasm” OR “Thymic malignancy”) |
| --- | --- | --- |
| 2 | disease stage | (Advanced OR Unresectable OR Metastatic OR Recurrent) |
| 3 | platinum-based chemotherapy | (Carboplatin OR Cisplatin OR Oxaliplatin OR Paclitaxel OR Docetaxel OR Doxorubicin OR Anthracyclines OR chemotherapy) |
| 4 | novel systemic therapies | ("targeted therapy" OR "targeted treatment" OR "targeted agent" OR “tyrosine kinase inhibitor” OR TKI OR crizotinib OR axitinib OR lenvatinib OR ramucirumab OR bevacizumab OR cetuximab OR "checkpoint inhibitor" OR "checkpoint blockade" OR "immune checkpoint therapy" OR immunotherapy OR Nivolumab OR Pembrolizumab OR Cemiplimab OR Toripalimab OR Sintilimab OR Camrelizumab OR Atezolizumab OR Durvalumab OR Avelumab OR Ipilimumab OR Tremelimumab) |
|  |  | #1, #2, #3 AND #4 |

1. PubMed

| 1 | thymic epithelial tumor | (“Thymic epithelial tumor” OR TET OR “Thymic carcinoma” OR “Thymoma” OR “Thymic tumor” OR “Thymic neoplasm” OR “Thymic malignancy” OR "Thymus Neoplasms"[Mesh]) |
| --- | --- | --- |
| 2 | disease stage | (Advanced OR Unresectable OR Metastatic OR Recurrent) |
| 3 | platinum-based chemotherapy | (Carboplatin OR Cisplatin OR Oxaliplatin OR "Platinum Compounds"[Mesh] OR Paclitaxel OR Docetaxel OR Doxorubicin OR Anthracyclines OR chemotherapy) |
| 4 | novel systemic therapies | ("targeted therapy" OR "targeted treatment" OR "targeted agent" OR “tyrosine kinase inhibitor” OR TKI OR crizotinib OR axitinib OR lenvatinib OR ramucirumab OR bevacizumab OR cetuximab OR "Molecular Targeted Therapy"[Mesh] OR "checkpoint inhibitor" OR "checkpoint blockade" OR "immune checkpoint therapy" OR immunotherapy OR Nivolumab OR Pembrolizumab OR Cemiplimab OR Toripalimab OR Sintilimab OR (Camrelizumab OR Atezolizumab OR Durvalumab OR Avelumab OR Ipilimumab OR Tremelimumab OR "Immune Checkpoint Inhibitors"[Mesh]) |
|  |  | #1, #2, #3 AND #4 |

**Table 3:** Outcome definitions.

| **Outcome** | **Formal Definition** |
| --- | --- |
| **Survival Outcomes** |  |
| Complete response (CR) | Disappearance of all target lesions. Any pathological lymph nodes (whether target or non-target) must have reduction in short axis to <10 mm. |
| Median duration of response (mDOR) | The duration from the onset of the first response to disease progression or death for any reason. |
| Objective response rate (ORR) | The proportion of patients with tumor size reduction of a predefined amount and for a minimum time period. |
| Overall survival (OS) | Time from randomization to death from any cause. |
| Partial response (PR) | At least a 30% decrease in the sum of diameters of target lesions, taking as reference the baseline sum diameters. |
| Progression-free survival (PFS) | The time from randomization until objective tumor progression or death, whichever occurs first. |
| Stable disease | Neither sufficient shrinkage to qualify for PR nor sufficient increase to qualify for PD, taking as reference the smallest sum diameters while on study. |
| **Safety and Tolerability**  **Outcomes** |  |
| Any-grade treatment-related adverse events  (TRAEs) | Any adverse event attributed to the study treatment (chemotherapy or ICI), graded 1 through 5 according to the National Cancer Institute's Common Terminology Criteria for Adverse Events (NCI-CTCAE). |
| Immune-related adverse events (irAEs) | Adverse events (AEs) related to the use of immune checkpoint inhibitor (ICI) therapy. |
| Grade ≥3 treatment-related  adverse events | Any adverse event attributed to the study treatment, graded as Grade 3 (severe), 4 (life-threatening), or 5 (death) according to the NCI-CTCAE. |

**Table 4.** Summary of findings and GRADE certainty of evidence assessment for primary outcomes.

|  | | | | | | | | | | |
| --- | --- | --- | --- | --- | --- | --- | --- | --- | --- | --- |
| **Certainty assessment** | | | | | | | **Summary of findings** | | | |
| **Participants (studies) Follow-up** | **Risk of bias** | **Inconsistency** | **Indirectness** | **Imprecision** | **Publication bias** | **Overall certainty of evidence** | **Study event rates (%)** | | **Impact** |  |
|  |  |  |  |  |  |  | **With No comparator (single arm study)** | **With Novel Systemic Agents** |  |  |
| **Median PFS** | | | | | | | | | | |
| 178 (6 non-randomized studies) | Serious ^a^ | Serious ^b^ | Not serious | Serious  ^c^ | Publication bias strongly suspected  ^d^ | ⨁◯◯◯ Very low  ^a,b,c,d^ | 16.22 months (95% CI 10.3 to 25.5) | | | |
| **Objective response rate (ORR)** | | | | | | | | | | |
| 208 (9 non-randomized studies) | Serious ^a^ | Serious  ^b^ | Not serious | Serious  ^c^ | Publication bias strongly suspected  ^d^ | ⨁◯◯◯ Very low  ^a,b,c,d^ | 57.7% (95% CI 44 to 70.3) | | | |
| **12-month PFS rate** | | | | | | | | | | |
| 178 (6 non-randomized studies) | Serious  ^a^ | Serious  ^b^ | Not serious | Serious  ^c^ | Publication bias strongly suspected  ^d^ | ⨁◯◯◯ Very low  ^a,b,c,d^ | 58.7% (95% CI 45.8 to 70.4) | | | |
| **12-month OS rate** | | | | | | | | | | |
| 122 (3 non-randomized studies) | Serious  ^a^ | Not serious | Not serious | Serious  ^c,e^ | Publication bias strongly suspected  ^e^ | ⨁◯◯◯ Very low  ^a,c,e^ | 96% (95% CI 84.8 to 99) | | | |

**CI:** confidence interval

#### Explanations

a. Serious risk of bias due to consistent confounding and additional methodological limitations across non-randomized studies.

b. Heterogeneity likely reflects clinical and methodological differences across studies, including treatment regimens, patient characteristics, and study design.

c. Confidence intervals were wide and the total sample size was limited, resulting in imprecision.

d. Given the limited number of studies and the non-comparative phase II design of most included trials, the presence of publication bias cannot be excluded.

e. Small total sample size.

**Figure 1.** Median progression-free survival (mPFS) (A) and subgroup analysis by drug class (B) and by prospective and retrospective studies (C).

C.I. confidence interval; Chi² (χ²), chi-squared test for heterogeneity; df, degrees of freedom; I², percentage of total variation across studies due to heterogeneity; ICI, immune checkpoint inhibitor; log[mPFS], logarithm of the median progression-free survival; mPFS, median progression-free survival; SE, standard error; Tau² (τ²), between-study variance.

**
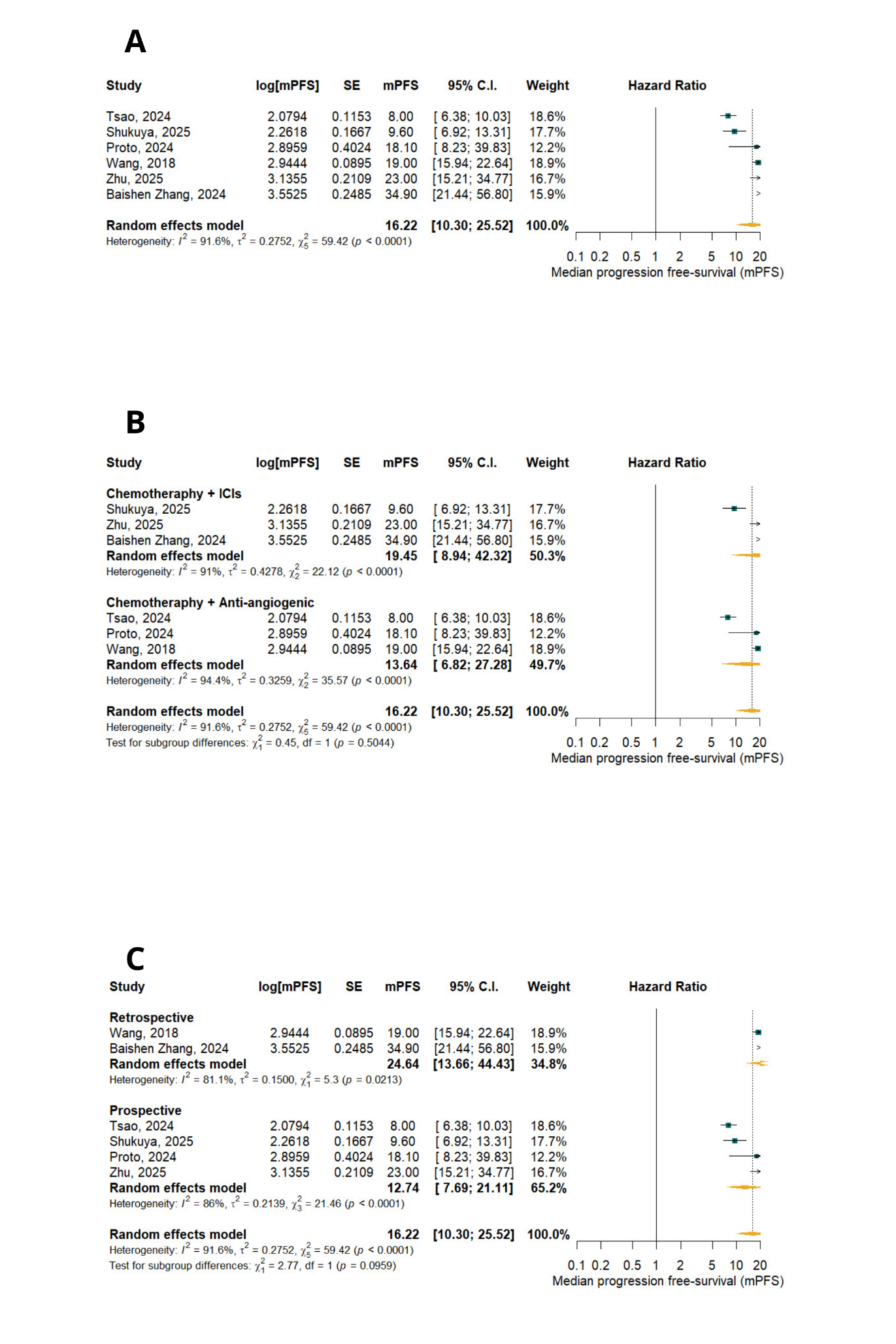
**

**Figure 2.** Median duration of response (mDOR).

C.I., confidence interval; Chi² (χ²), chi-squared test for heterogeneity; I², percentage of total variation across studies due to heterogeneity; log[mDOR], logarithm of the median duration of response; mDOR, median duration of response; SE, standard error; Tau² (τ²), between-study variance.


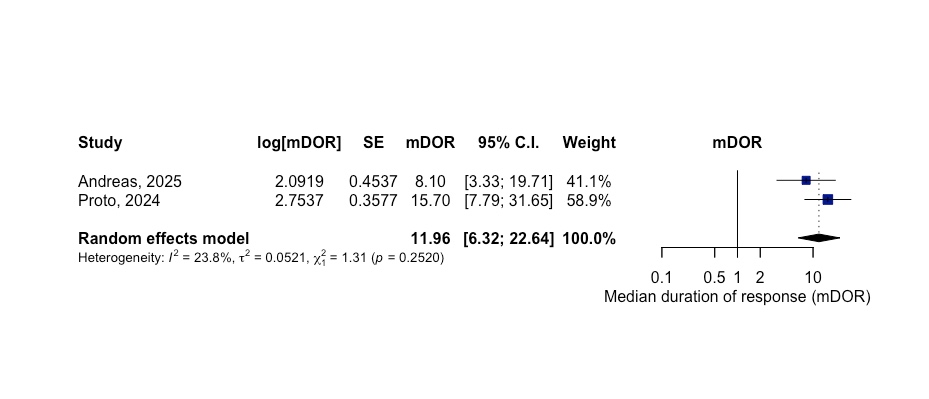


**Figure 3.** Complete response (CR).

CI, confidence interval; Chi² (χ²), chi-squared test for heterogeneity; df, degrees of freedom; I², percentage of total variation across studies due to heterogeneity; IV, inverse variance method; Tau² (τ²), between-study variance.


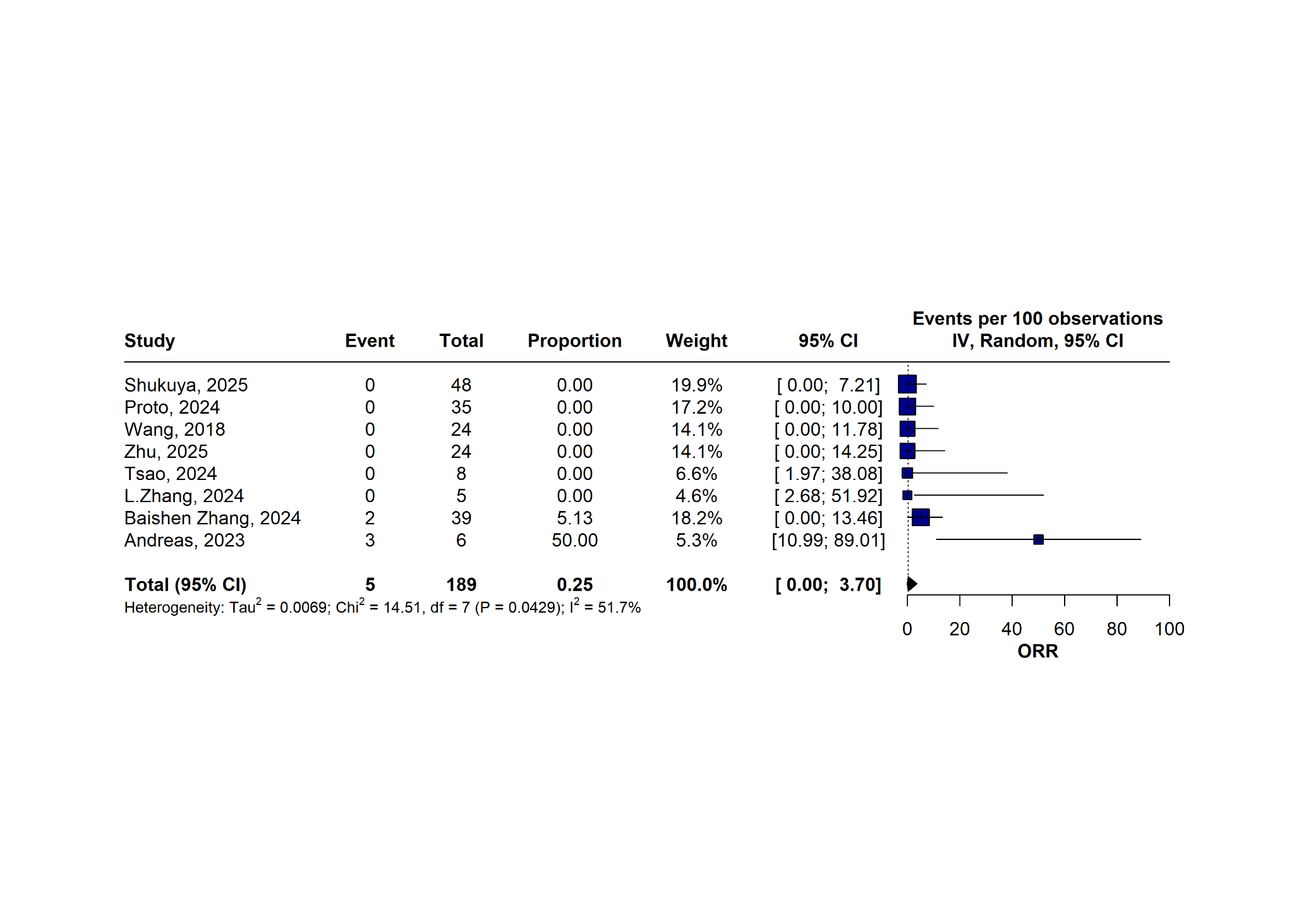


**Figure 4.** Partial response (PR) (A) and subgroup analysis by therapeutic regimen (B).

​​CI, confidence interval; Chi² (χ²), chi-squared test for heterogeneity; df, degrees of freedom; I², percentage of total variation across studies due to heterogeneity; IV, inverse variance method; Tau² (τ²), between-study variance.


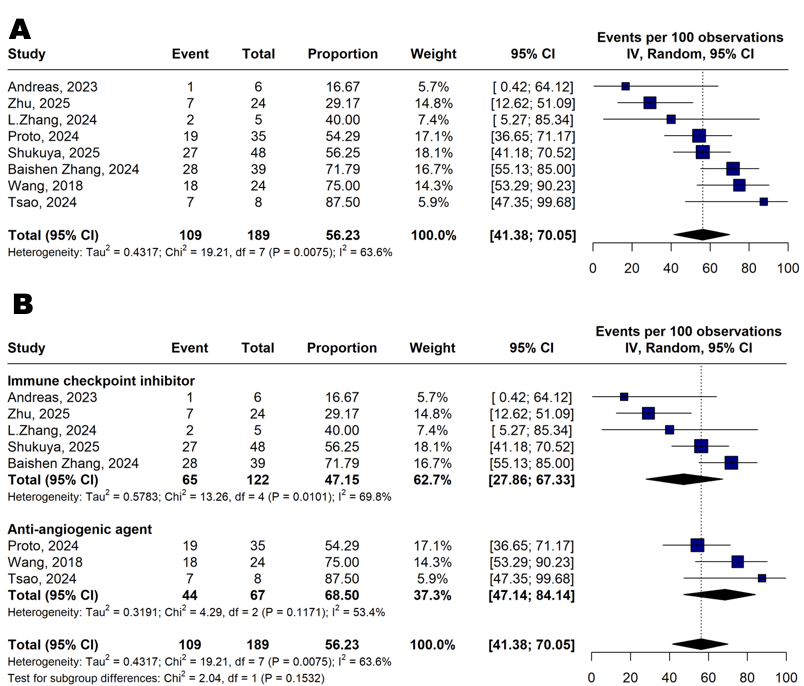


**Figure 5.** Stable disease (A) and subgroup analysis by therapeutic regimen (B).

CI, confidence interval; Chi² (χ²), chi-squared test for heterogeneity; df, degrees of freedom; I², percentage of total variation across studies due to heterogeneity; IV, inverse variance method; Tau² (τ²), between-study variance.


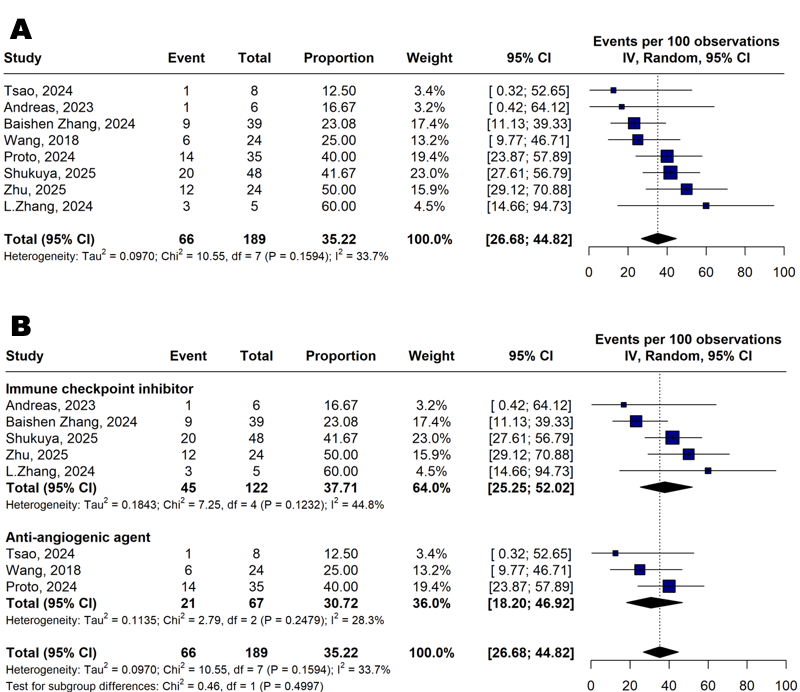


**Figure 6.** 12-month progression-free survival (PFS), subgroup analysis by drug class (A).

CI, confidence interval; Chi² (χ²), chi-squared test for heterogeneity; df, degrees of freedom; I², percentage of total variation across studies due to heterogeneity; IV, inverse variance method; PFS, progression-free survival; Tau² (τ²), between-study variance.


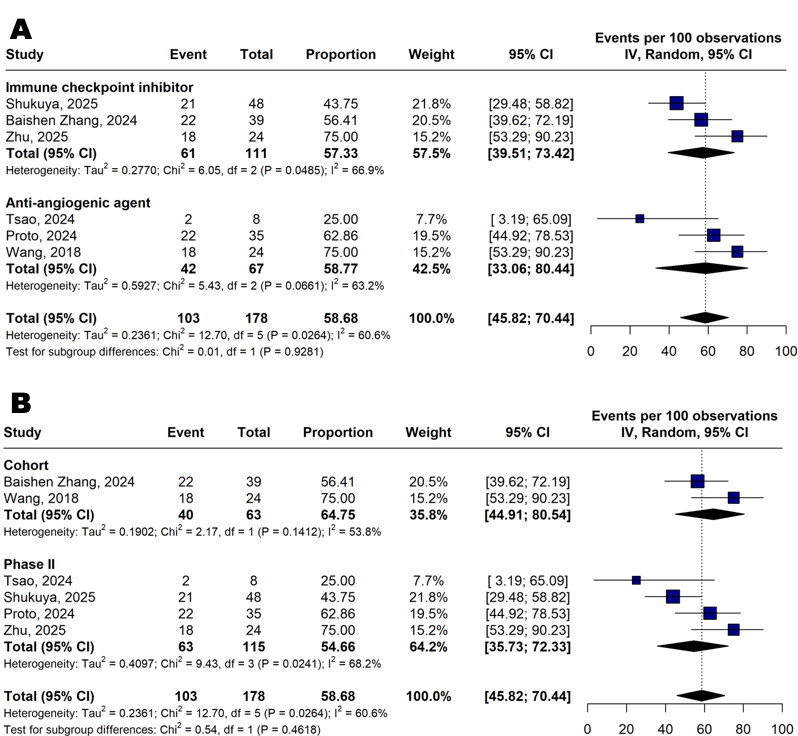


**Figure 7.** Leave-one-out sensitivity analysis for pooled median progression-free survival (mPFS).

**
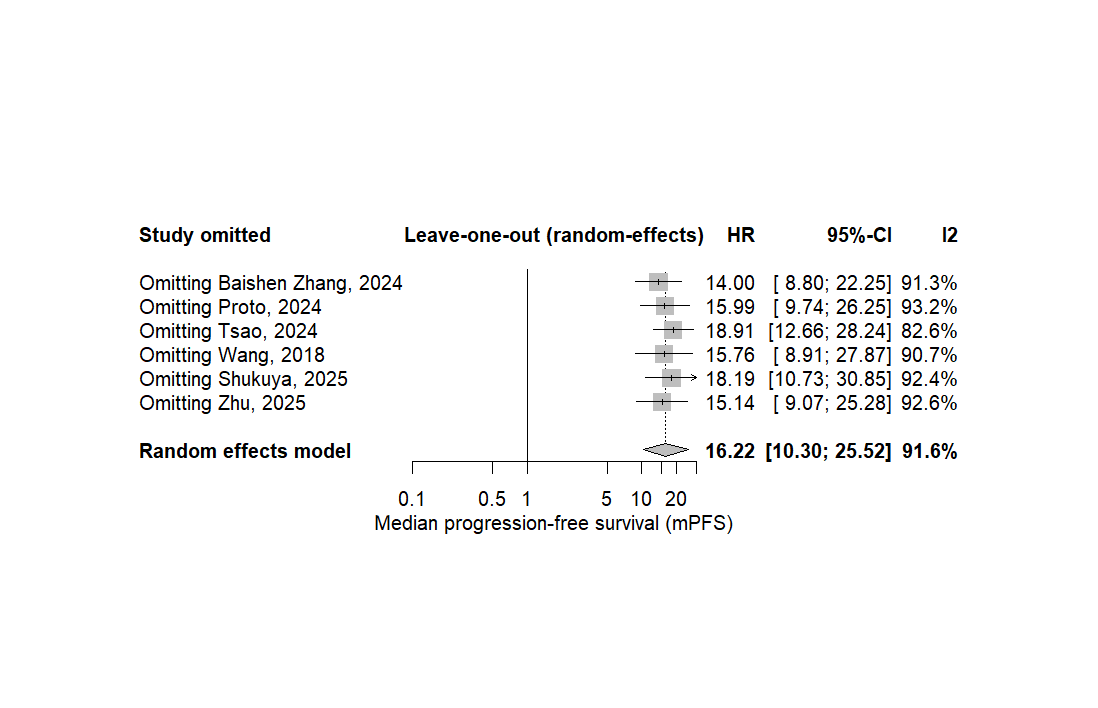
**

**Figure 8.** Leave-one-out sensitivity analysis of pooled median progression-free survival (mPFS) restricted to ICI-based regimens.


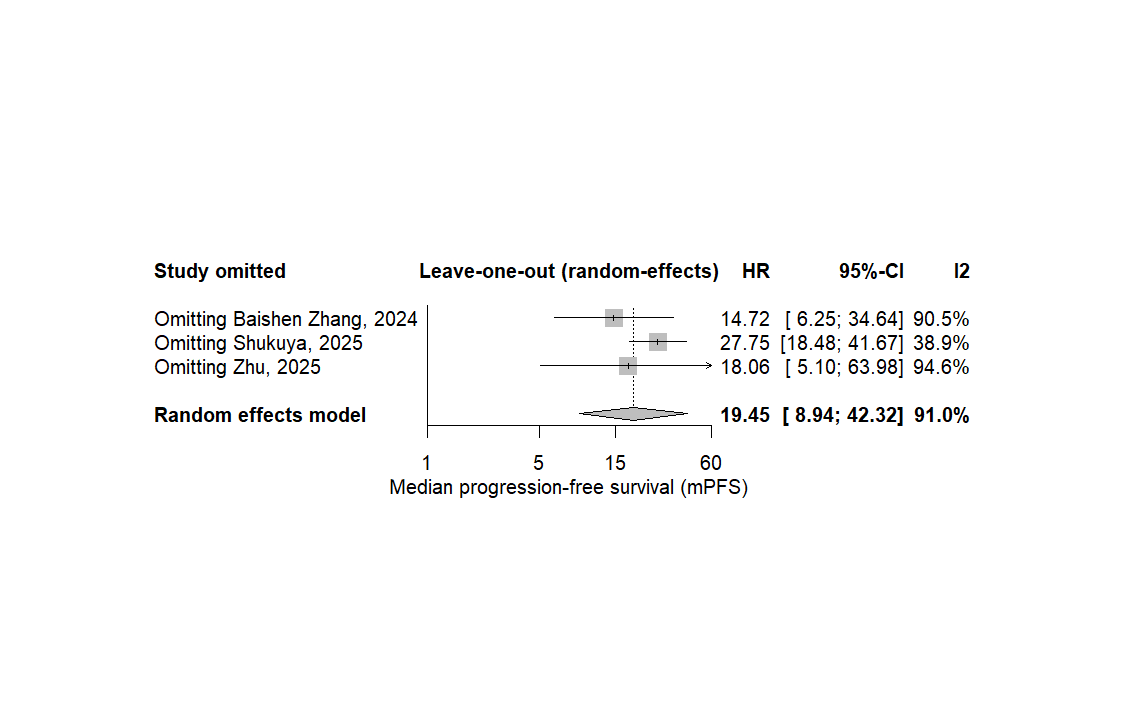


**Figure 9.** Most frequent any-grade treatment-related adverse events (TRAEs).

ALT: alanine aminotransferase.


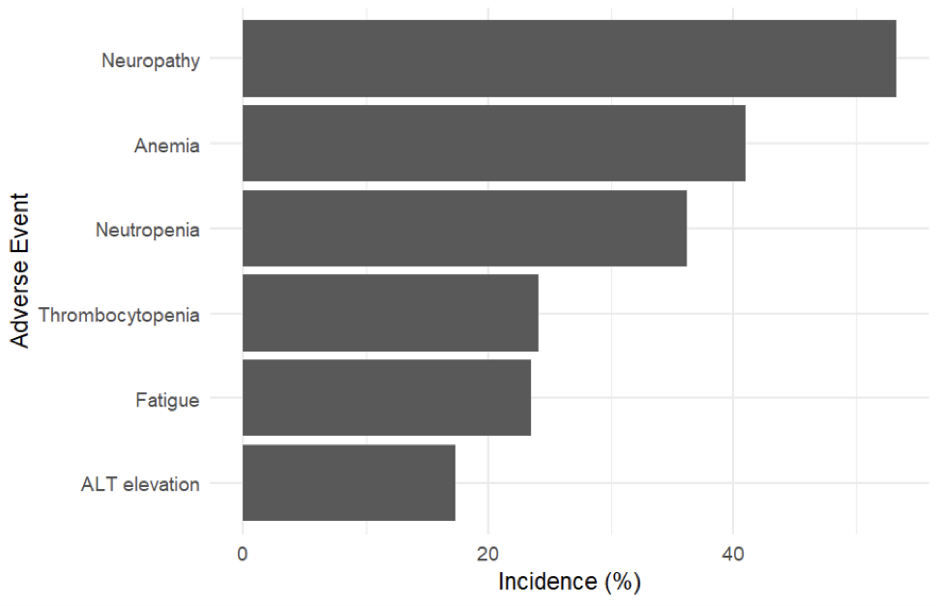


**Figure 10.** Treatment-related adverse events grade ≥ 3 subgroup analysis by therapeutic regimen.

CI, confidence interval; Chi² (χ²), chi-squared test for heterogeneity; df, degrees of freedom; I², percentage of total variation across studies due to heterogeneity; IV, inverse variance method; Tau² (τ²), between-study variance.

**
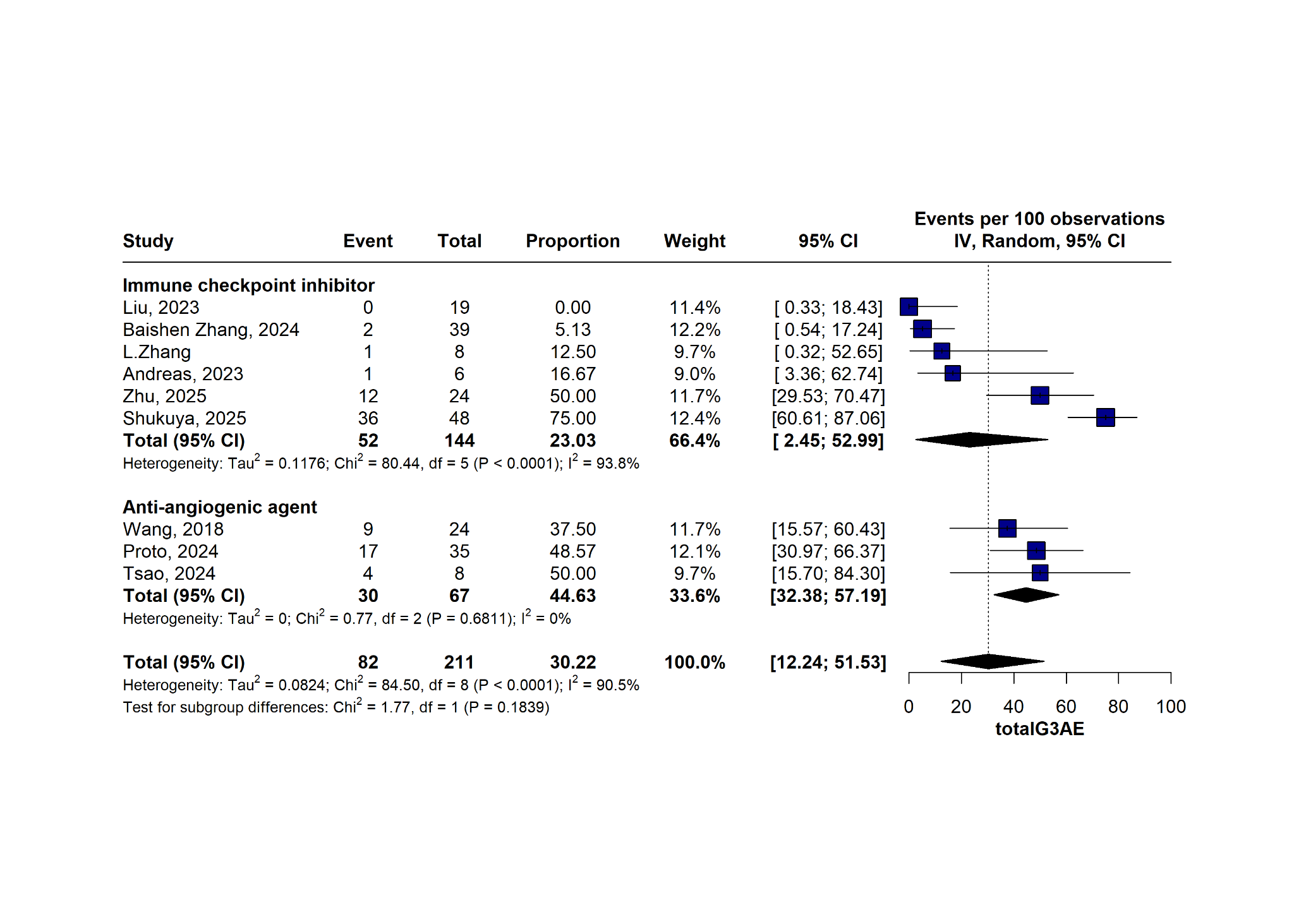
**

**Figure 11.** Treatment discontinuation due to toxicity (A). Subgroup analysis by therapeutic regimen (B).

CI, confidence interval; Chi² (χ²), chi-squared test for heterogeneity; df, degrees of freedom; I², percentage of total variation across studies due to heterogeneity; IV, inverse variance method; Tau² (τ²), between-study variance.


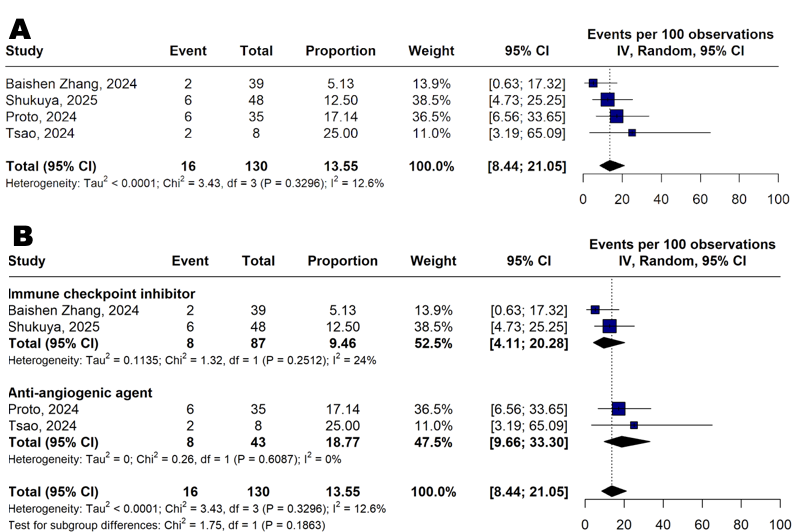


**Figure 12.** Immune-related any-grade treatment-related adverse events (irAEs) (A). Grade ≥ 3 (B).

CI, confidence interval; Chi² (χ²), chi-squared test for heterogeneity; df, degrees of freedom; I², percentage of total variation across studies due to heterogeneity; IV, inverse variance method; Tau² (τ²), between-study variance.

**
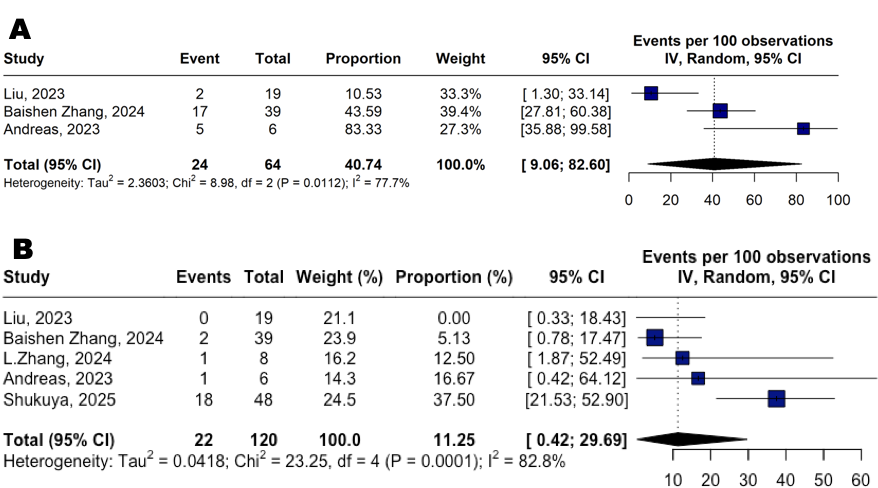
**

**Figure 13.** Risk of bias assessed by RoB-2 (A) and ROBINS-I (B).


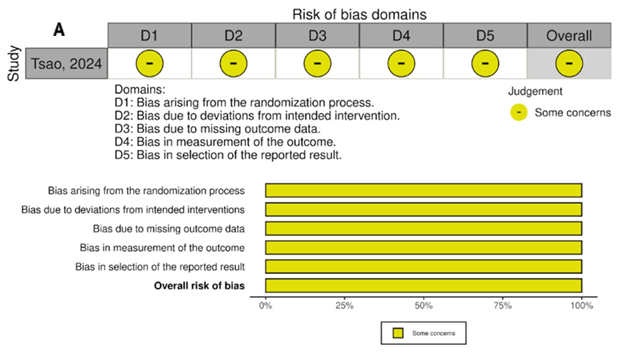


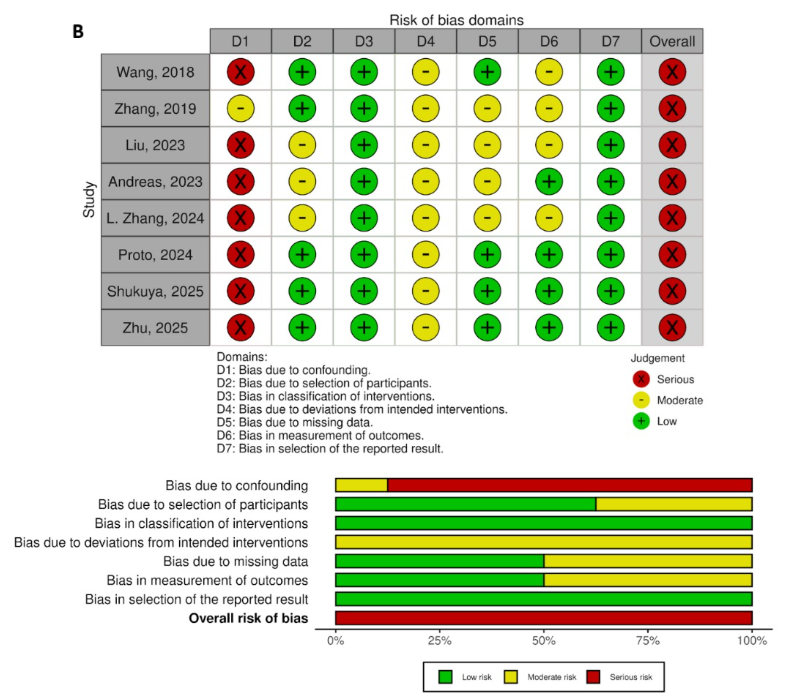

Supplement: Supplementary Material [file mmc1.docx]
